# The prevalence and associated factors of uncontrolled blood pressure in a rural community of Nepal: A cross-sectional study

**DOI:** 10.1371/journal.pgph.0005301

**Published:** 2026-01-08

**Authors:** Bihungum Bista, Sushmita Mali, Kjersti Mørkrid, Meghnath Dhimal, Biraj Man Karmacharya, Archana Shrestha

**Affiliations:** 1 Kathmandu University School of Medical Sciences (KUSMS), Dhulikhel, Kavre, Nepal; 2 Institute for Implementation Science and Health (IISH), Kathmandu, Nepal; 3 Division for Health Services, Norwegian Institute of Public Health (NIPH), Oslo, Norway; 4 Department of Global Public Health and Primary Care, University of Bergen, Bergen, Norway; 5 Nepal Health Research Council (NHRC), Kathmandu, Nepal; 6 Dhulikhel Hospital, Dhulikhel, Kavre, Nepal; The University of Adelaide, AUSTRALIA

## Abstract

Hypertension affects 1.28 billion adults globally, with two-thirds residing in low- and middle-income countries. In Nepal, about 60% of hypertensive individuals in urban settings have uncontrolled blood pressure, but the situation in rural settings is unknown. We aimed to determine the prevalence and factors associated with uncontrolled blood pressure among individuals diagnosed with high blood pressure, in a rural community in Nepal.We conducted a cross-sectional study among 1,008 individuals aged ≥30 years in Namobuddha Municipality, Nepal, who had a systolic blood pressure (SBP) ≥130 mmHg, a diastolic blood pressure (DBP) ≥85 mmHg, or were currently using antihypertensive medication. Participants were recruited through screening camps conducted in various parts of the study area. Demographic information, medical history, and lifestyle-related data were collected through face-to-face interviews, and anthropometric measurements were obtained. For analysis, uncontrolled blood pressure was defined as SBP ≥ 140 mmHg or DBP ≥ 90 mmHg. Descriptive statistics and multiple logistic regression analyses with 95% confidence intervals were conducted to assess the factors associated with uncontrolled blood pressure.About 64% of participants with diagnosed high blood pressure had uncontrolled blood pressure. After adjusting for age, gender, ethnicity, smoking, alcohol use, and physical activity, factors significantly associated with uncontrolled blood pressure included higher body mass index and poor adherence to antihypertensive medication. Specifically, each 1 kg/m² increase in BMI was associated with 7% higher odds of uncontrolled blood pressure (aOR: 1.07; 95% CI: 1.02–1.11), and adherence to antihypertensive medication lowered the odds of uncontrolled blood pressure (aOR: 0.21; 95% CI: 0.14–0.38).There is high prevalence of uncontrolled blood pressure among individuals diagnosed with high blood pressure in a rural setting. BMI, and poor adherence to antihypertensive medications are associated with uncontrolled high blood pressure. Targeted interventions to improve medication adherence and reduce BMI could enhance blood pressure control among hypertensive individuals in rural Nepal.

## Introduction

Non-communicable diseases (NCDs) constitute a significant global health burden, with hypertension identified as a major modifiable risk factor [1]. Uncontrolled blood pressure contributes to cardiovascular complications such as strokes, chronic kidney disease, and disability, ultimately diminishing the quality of life and increasing the risk of premature death [2,3]. Among individuals aged 40–89 years, each 20 mmHg increase in systolic blood pressure above 115 mmHg or 10 mmHg increase in diastolic blood pressure above 75 mmHg doubles the risk of major cardiovascular events and stroke [4]. Despite the availability of cost-effective strategies for hypertension prevention and control, it continues to pose a major public health challenge—accounting for an estimated 10.8 million deaths and 235 million Disability Adjusted Lifestyle Years (DALYs) annually. Moreover, while around 42% of adults with hypertension are diagnosed and receive treatment, only 21% achieve effective blood pressure control [1].

Generally factors associated with uncontrolled blood pressure include non-adherence to antihypertensive medication regimens, unhealthy diet including diets with high amount of salt,alcohol consumption, tobacco use, physical inactivity and sedentary lifestyle and being overweight or obese [5–7]. Additionally, demographic characteristics like sex and age, along with disease duration and comorbid health conditions, are linked to uncontrolled blood pressure [8–11]. Following global trends, risk factors like physical inactivity, alcohol intake, and high salt intake are increasing and fueling the burden of hypertension in Nepal [12,13]. Poor medication adherence, in addition to misinformation, disbelief, and myths about hypertension prevention and control strategies has also hindered in the control of hypertension [14]. The availability of trained healthcare workers, proper counselling from health workers, availability of essential medications, and logistical support also play a crucial role in hypertension control in Nepal [15,16].

A meta-analysis reported that only about 8% of individuals with diagnosed hypertension in Nepal have controlled blood pressure [17]. However, individual studies from urban settings—some of which were included in the meta-analysis—have reported higher control rates, with estimates of 17.2% in Surkhet, 13.8% in Kathmandu, 11.8% in Dhulikhel, and 15.8% in Pokhara [14,18–20]. Most available evidence originates from urban populations, where improved hypertension control may be associated with better access to healthcare and greater health awareness [21]. Individuals from rural and peri-urban areas, where approximately 82% of the country’s population live [22], remain underrepresented in the literature.

Thus, the epidemiology of hypertension, including management of hypertension within rural populations in Nepal remains inadequately described. This gap forces reliance on fragmented information and non-standardized interventions, hindering evidence-based planning and effective resource allocation. Consequently, to address this critical knowledge deficit and provide municipal health authorities with actionable insights into the determinants of hypertension control, an investigation of contextual factors influencing hypertension within rural settings is warranted. Therefore, we aimed to assess the prevalence and associated factors of uncontrolled blood pressure in a rural setting in Nepal.

## Materials and methods

### Ethics statement

The Ethical Review Board of the Nepal Health Research Council approved this study (Reference number: 459/2023). We obtained written informed consent from all participants. For illiterate participants, a trained study team member read the consent form aloud, and participants provided consent using a fingerprint in the presence of a witness. We did not provide financial incentives for participation.

### Study designs and settings

We analyzed population-based baseline cross-sectional data collected between August and November 2023 in Namobuddha Municipality to assess uncontrolled blood pressure as part of a Female Community Health Volunteer (FCHV)–led implementation study guided by the RE-AIM (reach, effectiveness, adoption, implementation, and maintenance) framework [23,24]. The RE-AIM framework evaluates interventions across five key dimensions: Reach (the proportion of the target population engaged), Effectiveness (impact on outcomes), Adoption (uptake by implementers), Implementation (fidelity and consistency), and Maintenance (sustainability over time). While the baseline data focus on estimating the prevalence of uncontrolled blood pressure, the original study aims to evaluate the implementation outcomes of the FCHV-led hypertension prevention and control strategy in Namobuddha municipality. Although Namobuddha is administratively classified as a municipality, it retains predominantly rural characteristics—such as dispersed settlements, limited health service availability, and agriculture-based livelihoods—making it comparable to other rural communities in Nepal [25].

### Study participants and recruitment

To identify eligible participants, we conducted 20 community-based screening camps on entire municipality in collaboration with local health facilities, where a total of 2,024 individuals were screened. Screening and assessment were conducted among all attendees, but this study focuses on the all the 1,008 participants who met the eligibility criteria. Thus, rather than sampling, we effectively conducted a complete assessment of all eligible participants identified during the community-based screening.

We excluded pregnant and postpartum women (up to eight weeks after birth) and individuals who were mentally incapacitated. Inclusion criteria were: (1) aged 30 years or older, (2) residing in Namobuddha Municipality for at least the past six months, and (3) either current use of antihypertensive medication or high blood pressure, defined as systolic blood pressure (SBP) ≥130 mmHg or diastolic blood pressure (DBP) ≥85 mmHg, confirmed by the health facility. Individuals with newly identified high blood pressure were referred for diagnosis and management.

### Data collection

Interviewers were trained about the data collection techniques conducted face-to-face interviews using modified standardized questionnaires, adapted from the WHO STEPS questionnaire (version 3.2), in Nepali. Responses were directly entered into a REDCap form.

The questionnaire included the following components:

**(a) Socio-demographic information**: gender (male/female), age (in years), ethnicity (brahmin/chhettri, janajati, dalit, others), marital status (unmarried, married, formerly married) educational status (illiterate/Basic/ primary/secondary/bachelor and higher), religion (hindu/buddhist/others).
**(b) Lifestyle information**: We defined smoking and alcohol use based on the WHO STEPS survey methodology [26,27]. We categorized tobacco use into current smokers (those who had smoked cigarettes within the past 30 days), past smokers (those who had smoked in the past but had quit and did not currently smoke), and non-smokers (those who had never smoked). We classified alcohol consumption into abstainers (those who had never consumed alcohol or had not consumed alcohol in their lifetime), non-current drinkers (consumed alcohol in the past but did not drink in the last 30 days (including those who drank within the past 12 months but not in the past 30 days))), and current drinkers (those who had consumed alcohol at least once in the past 30 days, regardless of frequency or quantity).

We assessed physical inactivity using the Global Physical Activity Questionnaire (GPAQ). To calculate total metabolic equivalent (MET) minutes per week, we multiplied the duration of each activity type by its corresponding MET value. We classified individuals as physically inactive if their total physical activity was less than 600 MET minutes per week [28]. We assessed adherence to dietary recommendations using the Global Dietary Recommendations (GDR) score. This score evaluates diet quality based on the consumption of nine health-protective food groups (NCD-Protect) and eight food groups to limit or avoid (NCD-Risk) over the past 24 hours. Scores range from 0 to 18, with higher scores indicating greater adherence to dietary recommendations [29].

**(c) Compliance to High Blood Pressure Treatment**: We used the Hill-Bone Compliance to High Blood Pressure Therapy (HB-HBP) Scale, a validated indirect method to assess adherence to hypertension therapy through self-report. This 14-item scale evaluates patient behaviors across three domains of hypertension treatment: Appointment Keeping (3 items), Diet [salt intake] (2 items), and Medication Adherence (9 items). The HB-HBP Scale is also validated in Nepalese context has a score range of 14–56, with higher scores indicating lower adherence to treatment [30,31]. For medication adherence, we classified participants who achieved the score of 14 as adherent, while those with scores above 14 were categorized as non-adherent. We also classified participants who reported not taking any antihypertensive medications as non-users.
**(d) Hypertension knowledge:** We assessed participants’ hypertension knowledge using the validated 22-item Hypertension Knowledge-Level Scale (HK-LS) [32]. The scale consists of six sub-dimensions: definition (2 items), medical treatment (4 items), drug compliance (4 items), lifestyle (5 items), diet (2 items), and complications (5 items). We scored each correct response 1 point, applying reverse scoring to 9 items. The total score ranged from 0 to 22, with higher scores indicating greater hypertension knowledge [32].
**(e) Social support:** We used the Multidimensional Scale of Perceived Social Support (MSPSS) to assess participants perceived social support. It scores from 1 to 7 and high score is considered as high perceived social support [33].
**(f) Anthropometric measurement**: We measured weight and height using standardized Seca equipment. We measured weight in kilograms with minimal clothing and without shoes, using a calibrated weighing machine, and recorded it to the nearest 0.1 kg. We measured height in centimeters without shoes using a stadiometer and recorded it to the nearest 0.1 cm. We calculated body mass index (BMI) as weight (kg) divided by height (m) squared.

### Blood pressure measurement

We measured blood pressure three times using a digital, automated blood pressure monitor (OMRON digital device, OMRON, Netherlands) with an appropriately sized cuff [27]. After each measurement, we allowed a 5-minute relaxation period. We defined hypertension based on the average of the last two measurements. Furthermore, we defined uncontrolled blood pressure as a systolic blood pressure of ≥140 mmHg or a diastolic blood pressure of ≥90 mmHg [34].

### Statistical analysis

For the purposes of analysis, participants were classified as new cases (those with no prior hypertension diagnosis who were identified during screening as having high blood pressure [SBP ≥ 130 mmHg or DBP ≥ 85 mmHg], further verified by health workers after referral) and old cases (those with a previous diagnosis of hypertension and/or currently on antihypertensive medication). Uncontrolled blood pressure was assessed separately in these groups. Age-specific distributions of systolic and diastolic blood pressure are presented in S1 Table. We conducted univariate and multivariable binary logistic regression analyses among previously diagnosed hypertensive individuals (i.e., old cases), excluding newly identified cases from screening camps who were subsequently confirmed by health workers. This approach was adopted because newly diagnosed individuals may not have had adequate time to initiate or adhere to antihypertensive treatment, potentially biasing associations with uncontrolled blood pressure. No data imputation was performed in this study. We conducted a complete case analysis for the primary outcome, including only participants with all three blood pressure measurements completed, i.e., 25 participants. For other variables, missing responses were retained in the dataset and clearly indicated in the descriptive tables. The extent of missing data for each variable is detailed in S2 Table. We did not exclude participants based on missing socio-demographic or risk factor information unless critical variables for specific analyses were missing. The extent of missing data for each variable is reported transparently. The covariates in the model were selected based on theoretical understanding and a directed acyclic graph (DAG) approach. The magnitude of association was presented as adjusted odds ratios (aORs) with 95% confidence intervals (CIs). We analyzed all data using STATA 15.

## Results

A total of 1008 participants with high blood pressure were included in this study. The average age of the participants was 59.1 (±13.4) years, and 48.5% were female (Table 1). The study population was characterized by low education levels (43.4% illiterate), predominantly Janjati (54.6%) ethnicity, high rates of marriage (80.2%) and Hindu affiliation (68.4%).

**Table 1:** Study participants’ characteristics (n = 1008).

Characteristics	Number	%
**Age(years)** mean ± SD	59.1 ± 13.4	
**Gender**		
Female	489	48.5
Male	519	51.5
Educational Status*		
Illiterate	437	43.4
Basic(Not completed primary)	209	20.8
Primary	148	14.7
Secondary	199	19.8
Bachelor and higher	14	1.4
**Ethnicity**		
Brahmin/Chhetri	391	38.8
Janjati	550	54.6
Dalit	67	6.6
**Marital status**		
Unmarried	16	1.6
Married	808	80.2
Formerly married	184	18.3
**Religion**		
Hindu	689	68.4
Buddhist	262	26
Others	57	5.7
**Family structure**		
Nuclear	584	57.9
Extended/Joint	424	42.1

*n=1 missing

Among new cases, 81.5% (n = 252) were uncontrolled, while 63.6% (n = 429) of old cases remained uncontrolled (Table 2). Uncontrolled individuals in both groups tended to be male (59.9% in new, 50.1% in old), Janjati (61.5% new, 56.2% old), and illiterate (38.9% new, 44.3% old). Most were currently married (82.1% new, 79.7% old). Mean age was 55.7 years in new uncontrolled and 59.1 years in old uncontrolled.

**Table 2:** Distribution of participant’s lifestyle and health characteristics (n = 983).

Characteristics	New cases (n = 309)		Old cases(n = 674)		Overall cases(n = 983)		Total (n = 983)
	Controlled (n = 57)	Uncontrolled (n = 252)	Controlled (n = 245)	Uncontrolled (n = 429)	Controlled (n = 302)	Uncontrolled (n = 681)	
**Age** (mean ± SD)	56.4 (14.97)	55.70 (13.94)	62.26 (12.76)	59.14 (12.75)	61.2 (13.37)	57.87 (13.39)	59.06 (13.39)
**Gender (n,%)**							
Female	25 (43.9)	101 (40.1)	138 (56.3)	214 (49.9)	163 (54.0)	315 (46.5)	489 (48.5)
Male	32 (56.1)	151 (59.9)	107 (43.7)	215 (50.1)	139 (46.0)	366 (53.7)	519 (51.5)
**Ethnicity** (n,%)							
Brahmin/Chhetri	21 (36.8)	82 (32.5)	118 (48.2%)	160 (37.3)	139 (46.0)	242 (35.5)	391 (38.8)
Janjati	32 (56.1)	155 (61.5)	107 (43.7%)	241 (56.2)	139 (46.0)	396 (58.1)	550 (54.6)
Dalit	4 (7.0)	15 (6.0)	20 (8.2%)	28 (6.5)	24 (7.9)	43 (6.3)	67 (6.6)
**Education (n,%)**							
Illiterate	24 (42.1)	98 (38.9)	113 (46.1)	190 (44.3)	137 (45.4)	288 (42.3)	437 (43.4)
Basic	11 (19.3)	42 (16.7)	61 (24.9)	88 (20.5)	72 (23.8)	130 (19.1)	209 (20.7)
Primary	5 (8.8)	48 (19.0)	28 (11.4)	64 (14.9)	33 (10.9)	112 (16.4)	148 (14.7)
Secondary and higher	17 (29.8)	64 (25.4)	43 (17.6)	87 (20.3)	60 (19.9)	151 (22.2)	213 (21.2)
**Marital status (n,%)**							
Currently Married	48 (84.2)	207 (82.1)	192 (78.4)	342 (79.7)	4(1.3)	12(1.8)	16(1.6)
Unmarried	1 (1.8)	8 (3.2)	3 (1.2)	4 (0.9)	240(79.5)	549(80.6)	808(80.2)
Formerly married	8 (14.0)	37 (14.7)	50 (20.4)	83 (19.3)	58(19.2)	120(17.6)	184(18.3)
**Smoking status (n,%)**							
Never	17 (30.8)	58 (23.3)	43 (17.6)	88 (20.7)	60(19.9)	146(21.4)	208(20.6)
Former	11 (19.6)	65 (26.1)	80 (32.7)	136 (31.9)	91(30.1)	201(29.5)	304(30.2)
Current	28 (50.1)	126 (50.6)	122 (49.8)	202 (47.4)	150(49.7)	328(48.2)	488(48.4)
**Alcohol Intake (n,%)**							
Never	31 (54.4)	119 (47.2)	150 (61.2)	221 (51.5)	181(59.9)	340(49.9)	533(52.9)
Non-current users	11 (19.3)	38 (15.1)	49 (20.0)	90 (21.0)	60(19.9)	128(18.8)	193(19.1)
Current intake (30 days)	15 (26.3)	95 (37.7)	45 (18.4)	118 (27.5)	60(19.9)	213(31.3)	280(27.8)
**Physical activity** (MET value)							
Insufficient	52 (91.2)	224 (88.9)	236 (96.3)	400 (93.2)	288(95.4)	624(91.6)	936(92.9)
Sufficient	5 (8.8)	28 (11.1)	9 (3.7)	29 (6.8)	14(4.6)	57(8.4)	72(7.1)
GDR score(mean ± SD)	10.87 (1.87)	10.41 (1.48)	11.08 (1.53)	10.74 (1.61)	11.04(1.60)	10.62(1.57)	10.74(1.59)
**Current medicine use** (n,%)							
Non user	53 (93.0)	240 (95.2)	30 (12.2)	168 (39.2)	83(27.5)	408(59.9)	501(49.7)
Yes but not adherent	3 (5.3)	5 (2.0)	120 (49.0)	147 (34.3)	123(40.7)	152(22.3)	284(28.2)
Yes and adherent	1 (1.8)	7 (2.8)	95 (38.8)	114 (26.6)	96(31.8)	121(17.8)	223(22.1)
BMI(**mean ± SD)**	24.43 (4.54)	24.91 (5.06)	25.46 (4.10)	26.51 (4.79)	25.27(4.20)	25.97(4.87)	25.76(4.67)
Hypertension knowledge(mean ± SD)	9.28 (2.87)	9.00 (3.01)	9.74 (2.83)	9.82 (2.71)	9.65(2.83)	9.51(2.85)	9.54(2.85)
Perceived social support(mean ± SD)	5.54 (0.87)	5.42 (0.88)	5.45 (0.93)	5.40 (0.85)	5.46(0.92)	5.41(0.86)	5.43(0.88)
**Total (n,%)**	57(18.4)	252(81.5)	245(36.5)	429(63.6)	302(30.7)	681(69.3)	983

Current smoking was reported by 50.6% of new and 47.4% of old uncontrolled cases. Recent alcohol intake (past 30 days) was higher in new uncontrolled (37.7%) than in old (27.5%). Physical inactivity was prevalent (88.9% new, 93.2% old). Only 2.8% of new uncontrolled were adherent to medication, compared to 26.6% in old cases. Mean BMI was 24.9 in new and 26.5 in old uncontrolled groups. Knowledge scores were similar (9.00 new vs. 9.82 old), and perceived social support was slightly lower in old (5.40 vs. 5.42). (Table 2)

Males had higher odds of uncontrolled blood pressure than females, though the association was not significant (crude Odds Ratio(cOR) 1.30, 95% CI: 0.95–1.78, *p* = 0.108) (Table 3). Older age was associated with lower odds (cOR 0.98, 95% CI: 0.97–0.99, *p* = 0.003). Compared to Brahmin/Chhetri, Janjati ethnicity had higher odds (cOR 1.66, 95% CI: 1.20–2.31, *p* = 0.003), while no significant difference was observed for Dalits. Medication use, whether adherent (cOR 0.21, 95% CI: 0.13–0.34) or non-adherent (cOR 0.22, 95% CI: 0.14–0.34), was linked to lower odds compared to non-use (*p* < 0.001). Current alcohol intake increased the odds (cOR 1.78, 95% CI: 1.20–2.68, *p* = 0.005). Sufficient physical activity showed higher, but non-significant, odds (cOR 1.90, 95% CI: 0.92–4.32, *p* = 0.100).

**Table 3:** Factors associated with uncontrolled blood pressure among patients diagnosed with hypertension, i.e., old cases (n = 674).

Characteristics	Level	cOR (95% CI)	p-value	aOR (95% CI)	p-value
Age		0.98 (0.97–0.99)	0.002**	0.99 (0.97–1.01)	0.372
Gender	Female	Ref		Ref	
	Male	1.30 (0.95–1.78)	0.107	1.13 (0.72–1.77)	0.595
Ethnicity	Brahmin/Chhetri	Ref		Ref	
	Janjati	1.66 (1.20–2.31)	0.002**	1.15 (0.75–1.75)	0.522
	Dalit	1.03 (0.56–1.94)	0.919	0.82 (0.39–1.71)	0.584
Education	Illiterate	Ref		Ref	
	Basic	0.86 (0.58–1.28)	0.454	0.76 (0.48–1.22)	0.257
	Primary	1.36 (0.83–2.27)	0.230	1.00 (0.55–1.85)	0.995
	Secondary	1.20 (0.78–1.87)	0.402	0.88 (0.48–1.64)	0.694
Marital status	Unmarried	Ref		Ref	
	Currently Married	1.34 (0.26–6.12)	0.706	1.32 (0.24–8.08)	0.744
	Formerly married	1.24 (0.24–5.87)	0.779	1.34 (0.23–1.64)	0.694
Smoking status	Never	Ref		Ref	
	Former	0.83 (0.52–1.31)	0.426	1.05 (0.63–1.75)	0.851
	Current	0.81 (0.52–1.24)	0.332	0.88 (0.53–1.45)	0.606
Alcohol Intake	Never	Ref		Ref	
	Intake last 12 months	1.25 (0.83–1.88)	0.286	1.10 (0.67–1.82)	0.714
	Current intake (30 days)	1.78 (1.20–2.68)	0.004**	1.18 (0.70–1.99)	0.528
Physical activity	Insufficient	Ref		Ref	
	Sufficient	1.90 (0.92–4.32)	0.100	1.34 (0.60–3.27)	0.493
GDR score		0.87 (0.79–0.96)	0.007**	0.90 (0.81–1.01)	0.081
Current Medicine Use	Non user	Ref		Ref	
	Yes but not adherent	0.22 (0.14–0.34)	0.000***	0.22 (0.13–0.35)	0.000***
	Yes and adherent	0.21 (0.13–0.34)	0.000***	0.23 (0.14–0.38)	0.000***
BMI		1.05 (1.02–1.09)	0.004**	1.07 (1.02–1.11)	0.003**
Perceived social support		0.94 (0.78–1.12)	0.477	0.90 (0.72–1.10)	0.300

*Ref: Reference*

Significance: *p < 0.05, **p < 0.01, ***p < 0.001

Higher BMI increased the odds (cOR 1.05, 95% CI: 1.02–1.09, *p* = 0.005), while higher GDR scores reduced them (cOR 0.87, 95% CI: 0.79–0.96, *p* = 0.008). Perceived social support was not associated with uncontrolled blood pressure (cOR 0.94, 95% CI: 0.78–1.12, *p* = 0.477).

After adjustment for age, gender, ethnicity, education, marital status, smoking, alcohol intake, physical activity, GDR score, BMI, and social support, medication use remained protective (adherent: adjusted Odds ratio(aoR): 0.23, 95% CI: 0.14–0.38, p < 0.001; non-adherent: aOR: 0.22, 95% CI: 0.13–0.35, p < 0.001). A per-unit increase in BMI increased odds (aOR: 1.07, 95% CI: 1.02–1.11, p = 0.002). A per-unit increase in GDR score showed a non-significant reduction (aOR: 0.90, 95% CI: 0.80–1.01, p = 0.077), while per-unit increases in age (aOR: 0.99, 95% CI: 0.97–1.01, p = 0.395) and perceived social support (aOR: 0.90, 95% CI: 0.73–1.10, p = 0.311) were non-significant.

## Discussion

This study aimed to assess the prevalence of uncontrolled blood pressure and their associated factors among individuals with a prior diagnosis of hypertension in Namobuddha Municipality, a rural area of Nepal. We found that nearly two-thirds (64%) of individuals diagnosed with high blood pressure had uncontrolled blood pressure. The key factors associated with uncontrolled blood pressure were a higher BMI and non-adherence to antihypertensive medication.

The prevalence of uncontrolled blood pressure in this rural population is higher than that reported in a recent study from an urban area of Nepal, where 55% of high blood pressure patients had uncontrolled blood pressure [35]. Urban residents may benefit from better access to health services and health education, which could contribute to improved control rates. Our findings are consistent with studies from rural India (63%) and Pakistan (70%) [36,37], highlighting the persistent challenge of managing hypertension in rural South Asian settings. Limited health literacy, poor awareness of hypertension risk factors, and constrained healthcare access may contribute to this burden [17,22].

Although Nepal has adopted the WHO Package of Essential Noncommunicable (PEN) Disease Interventions to improve primary healthcare delivery, the program remains largely facility-based and does not fully address community-level prevention and management activities [38]. Persistent health system challenges—such as inadequate counselling, long waiting times, and poor medication availability—likely exacerbate the problem of poor control of blood pressure in rural areas [17,22,39].

Our study identified a significant association between higher BMI and uncontrolled blood pressure, with each unit increase in BMI associated with a 5.6% increase in the odds of poor blood pressure control. This finding aligns with previous research [40–42] and reinforces the importance of integrating weight management into hypertension control strategies. While the link between higher body weight and uncontrolled blood pressure is well-recognized, even modest excess weight may contribute to challenges in blood pressure regulation [43,44]. In resource-limited rural settings, public health interventions that promote healthy eating and physical activity—delivered through existing community structures such as FCHVs—could help address this modifiable risk factor [45].

Medication adherence was also strongly associated with uncontrolled blood pressure. This finding supports existing evidence that antihypertensive therapy is effective when taken as prescribed [46]. In our context, non-adherence may reflect various barriers, including limited awareness, concerns about side effects, or affordability issues [47]. Conversely, individuals who adhere to treatment may have better access to care, stronger provider relationships, or more support from family and health workers [18,22]. These insights suggest the need for tailored interventions—such as counselling, reminder systems, or community-based follow-up—to promote adherence in rural populations to achieve the controlled blood pressure.

Dietary patterns are known to influence blood pressure, with diets rich in fruits, vegetables, and low in sodium helping reduce hypertension risk [6,48–53]. Although our unadjusted analysis showed a significant association between better diet quality and blood pressure control, this association was attenuated after adjustment for sociodemographic and behavioral variables. This may reflect either confounding or limited statistical power due to the modest sample size. Nonetheless, promoting healthy diets remains an essential component of hypertension control and should be emphasized in future community-based interventions.

In the adjusted analysis, we did not observe a statistically significant association between age and uncontrolled blood pressure, aligning with some studies [54,55] but contrasting with others reporting a positive association [13,36,56]. While crude analysis suggested a potential relationship, this may reflect greater non-adherence among younger adults, who often prefer lifestyle modification over pharmacological treatment [57–59]. The lack of significance in the adjusted analysis may also result from limited statistical power. These findings indicate the value of examining both crude and adjusted models when exploring complex behavioral outcomes like treatment adherence.

This study has several limitations. Its cross-sectional design prevents us from inferring causal relationships between exposures and uncontrolled blood pressure. Self-reported measures, such as medication adherence and lifestyle factors, may be subject to recall and social desirability biases. Additionally, the study’s focus on a single rural municipality limits generalizability to other areas with different population characteristics.

Despite these limitations, our study has notable strengths. We included a comprehensive sample of diagnosed hypertensive and high blood pressure individuals, which enhances representativeness. Standardized protocols were used for blood pressure and anthropometric measurements, improving the reliability of the data. Furthermore, we explored a wide range of potential associated factors, offering a holistic view of the factors influencing blood pressure control in a rural Nepalese setting.

## Conclusion

In conclusion, this study provides important information about the prevalence of uncontrolled blood pressure and its associated factors within a rural population in Nepal. The findings highlight the complex interplay of antihypertensive medication use, and weight management in hypertension management, which might need to include multifaceted and tailored interventions to improve blood pressure control. By addressing medication adherence, and healthy lifestyle behaviors, local health official can work towards reducing the burden of uncontrolled blood pressure and its associated morbidity and mortality in rural communities.

## Supporting information

S1 Table Age-wise distribution of SBP and DBP. (DOCX)

S2 Table Missing case analysis. (DOCX)
