# Supplementary material for: The prevalence and associated factors of uncontrolled blood pressure in a rural community of Nepal: A cross-sectional study
Source: PLOS Glob Public Health. 2026 Jan 8;6(1):e0005301. doi: 10.1371/journal.pgph.0005301 (PMC12782391; doi:10.1371/journal.pgph.0005301)
Supplement: S1 Table — (DOCX) [file pgph.0005301.s001.docx]

**S1 Table :** Age-wise distribution of SBP and DBP

| **Age group** | **SBP (mean, SD)** | **DBP (mean, SD)** |
| --- | --- | --- |
| **30-45** | 136.04, 13.44 | 95.26, 8.83 |
| **46-59** | 139.57, 15.43 | 94.87, 9.46 |
| **60 and above** | 141.18, 18.85 | 89.81, 11.42 |
