# Supplementary material for: The prevalence and associated factors of uncontrolled blood pressure in a rural community of Nepal: A cross-sectional study
Source: PLOS Glob Public Health. 2026 Jan 8;6(1):e0005301. doi: 10.1371/journal.pgph.0005301 (PMC12782391; doi:10.1371/journal.pgph.0005301)
Supplement: S2 Table — (DOCX) [file pgph.0005301.s002.docx]

**S2 Table : Missing case analysis**

| **Characteristics** | **Freq.** | **Percent/mean,SD** |
| --- | --- | --- |
| **Gender** |  |  |
| Female | 11 | 44 |
| Male | 14 | 56 |
| **Age (in years)** |  | 66.2, 11.25 |
| **Education** |  |  |
| illiterate | 12 | 50 |
| Basic | 7 | 29.17 |
| Primary | 3 | 12.5 |
| Secondary and higher | 2 | 8.33 |
| **Ethnicity** |  |  |
| Brahmin/Chhetri | 10 | 40 |
| Janjati | 15 | 60 |
| Total | 25 | 100 |
