# Supplementary material for: The prevalence and associated factors of uncontrolled blood pressure in a rural community of Nepal: A cross-sectional study
Source: PLOS Glob Public Health. 2026 Jan 8;6(1):e0005301. doi: 10.1371/journal.pgph.0005301 (PMC12782391; doi:10.1371/journal.pgph.0005301)
Supplement: Attachment — Submitted filename: Response to Reviewers.docx [file pgph.0005301.s004.docx]

**Dear Editor and reviewers,**

We sincerely thank the reviewers for their thorough and insightful feedback on our manuscript submitted to PLOS Global Public Health. Their comments have significantly strengthened the clarity, rigor, and quality of our work. Below, we address each comment point-by-point, detailing the revisions made to the manuscript. All changes have been incorporated into the revised manuscript, and we have ensured that the language is clear, grammatically correct, and adheres to standard English, as recommended. Following revisions were measured:

- **Study setting clarified** – Acknowledged Namobuddha’s administrative classification as an urban municipality but justified its rural characteristics; revisions made in the *Methods – Study setting* section.
- **Terminology standardized** – Replaced *“uncontrolled hypertension”* with *“uncontrolled blood pressure”* throughout the manuscript (title, results, and discussion) for consistency and accuracy.
- **Case definitions refined** – Clearly defined *new cases* (no prior diagnosis, identified during screening) and *old cases* (previously diagnosed or on medication) in the *Statistical Analysis* section.
- **Screening and sampling clarified** – Explained that screening was conducted among all 2,024 attendees, with analysis confined to 1,008 eligible participants; specified exclusions and described complete assessment approach.
- **Baseline data updated** – Provided characteristics of excluded participants (n=25) and included comparisons in supplementary files.
- **Methodological clarifications** – Corrected BP thresholds (130/85 mmHg for eligibility; 140/90 mmHg for uncontrolled BP definition), revised marital status categories, and added reference for community-based interventions via FCHVs.
- **Tables revised** – Corrected errors (removed redundant % symbol, clarified mean ± SD, merged small education categories, revised alcohol intake category).
- **Language editing** – Conducted a thorough grammar and language check by a native speaker and AI support; removed redundant/unclear sentences for clarity.

**Details response to reviewers**

**Response to Reviewers**

1. Does this manuscript meet PLOS Global Public Health’s [publication criteria](https://track.editorialmanager.com/CL0/https:%2F%2Fjournals.plos.org%2Fglobalpublichealth%2Fs%2Fcriteria-for-publication/1/010f01988000b27f-cdeafebf-ab6e-45b5-b00f-fdb466b1204c-000000/gtyJb3-yIS0jmJpsiQSmM_eC8VaZ5dyN7PtpVbERhpc=223)? Is the manuscript technically sound, and do the data support the conclusions? The manuscript must describe methodologically and ethically rigorous research with conclusions that are appropriately drawn based on the data presented.

Reviewer #1: Yes

Reviewer #2: Yes

2. Has the statistical analysis been performed appropriately and rigorously?

Reviewer #1: Yes

Reviewer #2: Yes

3. Have the authors made all data underlying the findings in their manuscript fully available (please refer to the Data Availability Statement at the start of the manuscript PDF file)?

The [PLOS Data policy](https://track.editorialmanager.com/CL0/https:%2F%2Fjournals.plos.org%2Fglobalpublichealth%2Fs%2Fdata-availability/1/010f01988000b27f-cdeafebf-ab6e-45b5-b00f-fdb466b1204c-000000/ExnOmuFE6qZdaet12NNf8FXOWM-FrD9mTBca97Y57rA=223) requires authors to make all data underlying the findings described in their manuscript fully available without restriction, with rare exception. The data should be provided as part of the manuscript or its supporting information, or deposited to a public repository. For example, in addition to summary statistics, the data points behind means, medians and variance measures should be available. If there are restrictions on publicly sharing data—e.g. participant privacy or use of data from a third party—those must be specified.

Reviewer #1: Yes

Reviewer #2: Yes

4. Is the manuscript presented in an intelligible fashion and written in standard English?

PLOS Global Public Health does not copyedit accepted manuscripts, so the language in submitted articles must be clear, correct, and unambiguous. Any typographical or grammatical errors should be corrected at revision, so please note any specific errors here.

Reviewer #1: Yes

Reviewer #2: Yes

5. Review Comments to the Author

Please use the space provided to explain your answers to the questions above. You may also include additional comments for the author, including concerns about dual publication, research ethics, or publication ethics. (Please upload your review as an attachment if it exceeds 20,000 characters)

**Reviewer #1: • In the Nepalese context, municipalities are generally classified as urban areas. However, the authors have conducted this study in Namobuddha Municipality to generate evidence relevant to rural settings. The authors need to justify classifying this setting as rural or revise the title, setting description, and relevant methodological details accordingly.**

*We thank the reviewer for this insightful comment. We agree that, administratively, municipalities in Nepal are classified as urban. Namobuddha Municipality was also declared an urban municipality during Nepal’s recent federal restructuring. However, despite its administrative status, Namobuddha retains predominantly rural characteristics. The majority of its population resides in dispersed settlements, livelihoods are largely agriculture-based, transportation and other infrastructure are limited, and access to healthcare services remains comparable to rural areas of Nepal.*

*To address the reviewer’s concern, we have clarified this point in the Methods – Study setting section by explicitly acknowledging its administrative classification as a municipality while justifying our description of it as having rural features. We also note that this situation is common across many newly declared municipalities in Nepal, which continue to function with rural socio-demographic and health system characteristics. Hence, we consider the findings of our study to be comparable and relevant to rural contexts in Nepal.*

**• In the third sentence of the introduction, the authors state: “Each incremental increase in systolic blood pressure (SBP) by 20 mmHg or more than 115 mmHg, or diastolic blood pressure (DBP) by 10 mmHg or more than 75 mmHg, doubles the risk of experiencing cardiovascular events and major stroke for 40-89 years.” It seems the intended meaning is that each 20 mmHg increase above 115 mmHg (SBP) and 10 mmHg increase above 75 mmHg (DBP) doubles the risk. However, the current phrasing is unclear and needs revision.**

*We thank the reviewer for pointing this out. We agree that our initial phrasing was unclear. The sentence has now been revised for clarity and accuracy.* The revised text reads as follows:

*“**Among individuals aged 40–89 years, each 20 mmHg increase in systolic blood pressure above 115 mmHg or 10 mmHg increase in diastolic blood pressure above 75 mmHg doubles the risk of major cardiovascular events and stroke*.”

**• In lines 79 to 82, the authors shift abruptly from discussing cost-effective interventions for NCDs to identifying hypertension as a major public health challenge. It would be clearer to focus the entire sentence specifically on hypertension.**

*We thank the reviewer for this helpful comment. We agree that the sentence shifted abruptly and could be made clearer by focusing specifically on hypertension. Accordingly, we revised the text to emphasize cost-effective interventions for hypertension rather than NCDs more broadly. The revised sentence now reads:*

*“Despite the availability of cost-effective strategies for hypertension prevention and control, it continues to pose a major public health challenge—accounting for an estimated 10.8 million deaths and 235 million DALYs annually.”*

• **The authors mention conducting screening camps at the community level, intending to screen all individuals visiting the health facilities regardless of BP level. However, they specify BP thresholds in the eligibility criteria. If screening was conducted among all visitors but the analysis was limited to those meeting the eligibility criteria, the text should be revised accordingly. For instance: “Screening was conducted among all participants, but this analysis is confined to 1,008 participants meeting the eligibility criteria.”**

*We thank the reviewer for this insightful comment. We agree that our original description could have implied that screening was limited only to those with high blood pressure. We have revised the text to clarify that screening was conducted among all 2,024 attendees of the 20 community-based camps, but this study focuses on the 1,008 participants who met the eligibility criteria. This revision clearly distinguishes between the overall screening effort and the subset of participants included in the study population.*

The revised sentences read as:

“*To identify eligible participants, we conducted 20 community-based screening camps on entire municipality in collaboration with local health facilities, where a total of 2,024 individuals were screened. Screening and assessment were conducted among all attendees, but this study focuses on the all the 1,008 participants who met the eligibility criteria. Thus, rather than sampling, we effectively conducted a complete assessment of all eligible participants identified during the community-based screening.*

*We excluded pregnant and postpartum women (up to eight weeks after birth) and individuals who were mentally incapacitated. Inclusion criteria were: (1) aged 30 years or older, (2) residing in Namobuddha Municipality for at least the past six months, and (3) either current use of antihypertensive medication or high blood pressure, defined as systolic blood pressure (SBP) ≥130 mmHg or diastolic blood pressure (DBP) ≥85 mmHg, confirmed by the nearest health facility.*

*We obtained the written informed consent from all participants prior to the interview and for………..”*

• **In line 139, the authors list both ‘married’ and ‘ever married’ as marital status categories. These are overlapping, as ‘married’ falls under ‘ever married.’ This should be corrected. If ‘ever married’ is intended to refer to divorced or separated individuals, it should be specified clearly.**

*We thank the reviewer for highlighting this issue. We agree that listing both “married” and “ever married” as separate categories is overlapping. In our revised text, we have clarified the marital status categories as: “unmarried,” “currently married,” and “formerly married” (including divorced, separated, and widowed individuals) to avoid ambiguity and made necessary correction in table and text. The corrected description now reads:*

*“marital status (**unmarried, married, formerly married)).”*

• **The phrase “Physical measurements included the following components” is unnecessary, as the measurements are listed sequentially and have not been categorized in earlier sections. This sentence can be removed.**

*We thank reviewer for pointing out this issue and we have removed that sentence from the manuscript.*
**• In Table 1, the percentage symbol (%) is not needed in the last column under the ethnicity variable and should be removed.**

*Thank you reviewer pointing out this typo, and we have corrected in the manuscript.*
• **In line 221, the authors state that 81.5% of new cases were uncontrolled, implying that the remaining were controlled. However, it is unclear how 'new cases' were defined. Individuals with controlled BP and no prior diagnosis would be considered normotensive, not new cases. This needs clarification.**

*We thank the reviewer for this important observation. We agree that the definition of “new cases” was not clearly described. In our study,* ***new cases*** *refer to individuals who were identified during the screening camps as having elevated blood pressure (SBP ≥130 mmHg or DBP ≥85 mmHg) and were further verified by health workers after referral as having high blood pressure, with no prior diagnosis or treatment for hypertension. Participants whose BP was below these thresholds and had no prior diagnosis were considered normotensive and were therefore not included in this analysis of 1,008 participants.*

*The definitions of* ***new cases*** *and* ***old cases*** *have been included in the Statistical Analysis subsection, where they are most relevant. The updated paragraph reads:*

*“**We identified 1,008 cases of high blood pressure and analyzed data from 983 participants who had complete sets of three blood pressure readings. For the purposes of analysis, participants were classified as* ***new cases*** *(those with no prior hypertension diagnosis who were identified during screening as having elevated blood pressure [SBP ≥130 mmHg or DBP ≥85 mmHg], further verified by health workers after referral) and* ***old cases*** *(those with a previous diagnosis of hypertension and/or currently on antihypertensive medication). Uncontrolled hypertension was assessed separately in these groups.”*

**• Also in line 221, the authors mention that “healthy behaviours were sub-optimal.” Unless an index variable for ‘healthy behaviour’ was created, this sentence should be omitted. The authors can instead present the findings for individual behavioural variables.**

*Thank you reviewer for mentioning this, as suggested we omitted the “healthy behaviours were sub-optimal.” statement.*
**• In Table 2, the authors should specify that the numbers represent mean and standard deviation. This can be done using parentheses alongside each variable name (e.g., “Age (mean ± SD)”).**

*Thank you, reviewer for suggesting this, as suggested we inserted the “*(mean ± SD)”*.*
**• It appears that some cell values are limited due to small sample sizes under educational status in Table 2. Unless clinically justified, the authors may consider merging 'secondary' and 'bachelor’s and above' into a single category: ‘secondary and above.’**

*Thank you reviewer for noticing this .We have made the necessary correction as per the reviewer suggestion in table and text.*
• **In Table 2, the category ‘in last 12 months’ under alcohol intake is not self-explanatory. To improve clarity, it should be revised to ‘intake in the last 12 months.’**

*Thank you, reviewer for suggesting this, as suggested we revised the text*”*.*
• **A thorough grammar check is recommended throughout the manuscript.**

*Thank you, reviewer, for noticing the inconsistencies in grammer . We have corrected the English with native speaker, and took the artificial intelligence support on that part. We hope , this will help to maintain the grammatical rigour in our paper.*

**Reviewer #2: Thank you for the opportunity to review the paper. This is an important paper. However, I have following suggestion to improve rigor and quality of the paper:**

**1. Can you explain the sampling technique for this study?**

*Thank you for pointing out the lack of clarity regarding the sampling procedure. We have now revised the methodology section to provide more detailed information on participant recruitment. Specifically, we clarified that we screened all individuals attending the 20 community-based screening camps, and all eligible participants (n = 1,008) were included in the study.*

*We have added following statement in the methodology section, that:*

*“Screening and assessment were conducted among all attendees, but this study focuses on the all the 1,008 participants who met the eligibility criteria. Thus, rather than sampling, we effectively conducted a complete assessment of all eligible participants identified during the community-based screening”*

**2. What is the definition of uncontrolled hypertension? The paper at the moment looks like an analysis of uncontrolled blood pressure rather than the prevalence of uncontrolled hypertension prevalence. I presume they are different given uncontrolled hypertension considers persistent raised BP even after pharmacological or lifestyle intervention.**

*Thank you for this insightful comment. We agree that the terminology required clarification. In our manuscript, we had used both “uncontrolled hypertension” and “uncontrolled blood pressure.” To avoid confusion and ensure consistency, we have now adopted the term* ***“uncontrolled blood pressure” uniformly throughout the manuscript****. This terminology appropriately encompasses both previously diagnosed individuals (“old cases”) and those newly identified during our screening who had not yet entered the care pathway. Accordingly, we have revised the title, results, and discussion sections to reflect this change.*

3. **Detailed baseline characteristics of the missing and excluded cases are needed in the result section. Was there any difference in their characteristics?**

*Thank you,reviewer for the generous comment. Out of 1,008 participants, we excluded 25 from the detailed analysis because of missing values in the last two blood pressure readings out of the required three. We did not include these participants in the inferential statistics but retained them in the description of baseline characteristics. When we compared key socio-demographic variables (gender, ethnicity, and education status), we found no meaningful differences between excluded and included participants. We did observe a difference in mean age, but we assume this is unlikely to distort the main analysis. We provide detailed information on the missing cases in the supplementary file.Additionally, we have included about the 25 missing cases in statistical sub-section as well.*

4. **Can you also specify the mean blood pressure by age group?**
*Thank you reviewer for this valuable suggestion. We analyzed both systolic and diastolic blood pressure by age group and have included this information in the supplementary file. Since the main focus of our paper is on controlled versus uncontrolled blood pressure, we believe that including mean blood pressure by age group in the main text may not add substantial value to our primary objectives. However, we have made the information available in the supplementary material for completeness.*
5**. It is also unclear if the paper is focusing on lifestyle medication or pharmacological control of hypertension.**

*Thank you for your feedback. We have clarified the definitions of new and previously diagnosed cases, which should help readers understand the intention of our paper. As our analysis is limited to previously diagnosed cases, this paper considers both pharmacological and lifestyle control of blood presssure, consistent with the definition of uncontrolled blood pressure (BP ≥140/90 mmHg, with or without medication ).*

**6. How are new cases assessed for medication adherence? What is the definition of new cases?**

*In our study, new cases refer to individuals who had not been previously identified as having high blood pressure by a health worker prior to our screening. After being identified during screening camp conducted by us, they were referred to health facilities for confirmation and management if required. Those confirmed by a health worker and who provided consent were then recruited into the study and interviewed. Since the interval between screening and the detail interview was approximately one month, we were also able to assess medication adherence among newly diagnosed individuals using the Hill-Bone Medication Adherence Scale, though number of individuals was too small.*
**7. The cut off points and definition for hypertension differs in line 123- 126, and line 186: Blood pressure measurement.**

*Thank you for pointing this out. We would like to clarify that the definitions in line 123–126 and line 186 refer to two different contexts. The threshold of 130/85 mmHg (line 123–126) was used as the eligibility criterion for study inclusion, ensuring that individuals at risk were captured during screening. In contrast, the threshold of 140/90 mmHg (line 186) refers to the standard definition of uncontrolled blood pressure, which was applied for outcome assessment.*

8. **Need references to this statement: In resource-limited rural settings, 295 public health interventions that promote healthy eating and physical activity—delivered through 296 existing community structures such as Female Community Health Volunteers (FCHVs)—could 297 help address this modifiable risk factor**

*Thank you reviewer, we have included the reference.*

9. Enrolment into Medication and medication adherence are two different variables. Simply enrolling in medication can be a predictor for blood pressure control. So, would suggest to disintegrate as two variables after the collinearity check

*We appreciate the reviewer’s suggestion and fully acknowledge that medication enrollment and medication adherence are conceptually distinct. However, in our context, considering them separately posed analytical challenges. If we restrict the analysis to medication adherence alone, this would limit the sample only to those already on medication, thereby substantially reducing the dataset for regression analysis and resulting in imprecise estimates. On the other hand, considering only medication use would overlook the critical role of adherence, which is well established in the literature as an essential determinant of hypertension control.*

*To address both situations, we created a* ***composite variable combining medication use and adherence****. This approach allowed us to capture the broader influence of treatment behavior while maintaining statistical power and more stable estimates, despite recognizing the conceptual difference between the two constructs.*

**10. I would recommend a thorough review of the term “uncontrolled hypertension” as this paper basically focuses on the prevalence of hypertension or “uncontrolled blood pressure”. The discussion section should be appropriated accordingly. revise my response: "Thank you for this thoughtful comment.**
*As noted in response to comment 2, we have adopted “uncontrolled blood pressure” throughout the manuscript, revising the title, results, and discussion to align with this terminology for consistency and clarity.*

6. PLOS authors have the option to publish the peer review history of their article ([what does this mean?](https://track.editorialmanager.com/CL0/https:%2F%2Fjournals.plos.org%2Fglobalpublichealth%2Fs%2Feditorial-and-peer-review-process%23loc-peer-review-history/1/010f01988000b27f-cdeafebf-ab6e-45b5-b00f-fdb466b1204c-000000/QYK_uHEu9mbCx41O3pGMHj4IZdV23xGzukBAe3a7EK0=223)). If published, this will include your full peer review and any attached files.


**Do you want your identity to be public for this peer review?** If you choose “no”, your identity will remain anonymous but your review may still be made public.


For information about this choice, including consent withdrawal, please see our [Privacy Policy](https://track.editorialmanager.com/CL0/https:%2F%2Fwww.plos.org%2Fprivacy-policy/1/010f01988000b27f-cdeafebf-ab6e-45b5-b00f-fdb466b1204c-000000/P8srXXA5B3K9p2HIzzFl6Cx9mzSJkfdf-yiKp45v7RQ=223).

Reviewer #1: No

Reviewer #2: No

We believe these revisions address all reviewer concerns and enhance the manuscript’s clarity, rigor, and scientific quality. We are grateful for the opportunity to improve our work and look forward to your feedback. Please let us know if further revisions are needed.

Sincerely

Bihungum Bista

Corresponding author
