# Supplementary material for: The prevalence and associated factors of uncontrolled blood pressure in a rural community of Nepal: A cross-sectional study
Source: PLOS Glob Public Health. 2026 Jan 8;6(1):e0005301. doi: 10.1371/journal.pgph.0005301 (PMC12782391; doi:10.1371/journal.pgph.0005301)
Supplement: Attachment — Submitted filename: Response_to_Reviewers_auresp_2.docx [file pgph.0005301.s005.docx]

**Dear Editor and Reviewers,**

We sincerely thank Reviewer #3 for the thorough, constructive, and insightful feedback on our manuscript. The comments have greatly improved the clarity, consistency, and methodological rigor of the revised version. We have carefully addressed each point and incorporated all recommended revisions into the manuscript. Below, we provide a point-by-point response summarizing the changes made:

**Summary of Revisions Implemented in Response to Reviewer #3**

- **Clarified abstract phrasing** – Revised the final sentence for clarity by stating:
  *“Adherence to antihypertensive medication lowered the odds of uncontrolled blood pressure (aOR: 0.21; 95% CI: 0.14–0.38).”*
- **Standardized terminology** – Ensured consistent use of terms such as *uncontrolled blood pressure*, *high blood pressure*, and *basic education*. Clarified the appropriate use of “compliance” versus “adherence” based on terminology used in the Hill-Bone Scale and HKLS.
- **Corrected and standardized abbreviations** – All abbreviations (e.g., DALY, BMI) are now defined at first mention and used consistently throughout the manuscript.
- **Addressed typographical issues** – Corrected spacing and minor formatting errors (e.g., “include non-”).
- **Expanded description of the RE-AIM framework** – Added a concise explanation of the five RE-AIM dimensions and clarified how the framework guided the FCHV-led hypertension study.
- **Justified the study setting** – Cited the *Degree of Urbanization* report to explain why Namobuddha Municipality, despite its administrative classification, exhibits predominantly rural characteristics relevant to this study.
- **Clarified hypertension confirmation procedures** – Removed the term “nearest” to reflect that participants were included irrespective of where their diagnosis was confirmed (including Kathmandu or Dhulikhel).
- **Revised description of interviewer training** – Replaced subjective language with a factual description:
  *“Interviewers received 2 days of training covering data collection techniques, face-to-face interview methods, and use of WHO STEPS–adapted tools.”*
- **Refined smoking and alcohol definitions** – Clarified that smoking status refers specifically to cigarette smoking; revised alcohol consumption categories into *abstainers*, *non-current drinkers*, and *current drinkers* for clarity.
- **Specified reporting of GDR scores** – Indicated that the GDR score is presented as *mean ± standard deviation (SD)*.
- **Ensured consistent education terminology** – Standardized all references to “basic education” when referring to education below the primary level.

**Reviewer's Responses to Questions**

**Comments to the Author**

1. If the authors have adequately addressed your comments raised in a previous round of review and you feel that this manuscript is now acceptable for publication, you may indicate that here to bypass the “Comments to the Author” section, enter your conflict of interest statement in the “Confidential to Editor” section, and submit your "Accept" recommendation.

Reviewer #3: All comments have been addressed

2. Does this manuscript meet PLOS Global Public Health’s [publication criteria](https://track.editorialmanager.com/CL0/https:%2F%2Fjournals.plos.org%2Fglobalpublichealth%2Fs%2Fcriteria-for-publication/1/010f019a9f54f1c7-26fceb9f-7126-45f4-8650-44e2e5defc4b-000000/QDL_AMSAoFanEE5Txyfaf1ZLAAB9J3rSF2QIT-sw_iM=238)? Is the manuscript technically sound, and do the data support the conclusions? The manuscript must describe methodologically and ethically rigorous research with conclusions that are appropriately drawn based on the data presented.

Reviewer #3: Yes

3. Has the statistical analysis been performed appropriately and rigorously?

Reviewer #3: Yes

4. Have the authors made all data underlying the findings in their manuscript fully available (please refer to the Data Availability Statement at the start of the manuscript PDF file)?

The [PLOS Data policy](https://track.editorialmanager.com/CL0/https:%2F%2Fjournals.plos.org%2Fglobalpublichealth%2Fs%2Fdata-availability/1/010f019a9f54f1c7-26fceb9f-7126-45f4-8650-44e2e5defc4b-000000/DF6VfwJUGLYjyTNqNALC1BdNnbzccN64iDjrZXHwk-8=238) requires authors to make all data underlying the findings described in their manuscript fully available without restriction, with rare exception. The data should be provided as part of the manuscript or its supporting information, or deposited to a public repository. For example, in addition to summary statistics, the data points behind means, medians and variance measures should be available. If there are restrictions on publicly sharing data—e.g. participant privacy or use of data from a third party—those must be specified.

Reviewer #3: Yes

5. Is the manuscript presented in an intelligible fashion and written in standard English?

PLOS Global Public Health does not copyedit accepted manuscripts, so the language in submitted articles must be clear, correct, and unambiguous. Any typographical or grammatical errors should be corrected at revision, so please note any specific errors here.

Reviewer #3: Yes

6. Review Comments to the Author

Please use the space provided to explain your answers to the questions above. You may also include additional comments for the author, including concerns about dual publication, research ethics, or publication ethics. (Please upload your review as an attachment if it exceeds 20,000 characters)

Reviewer #3: Feedback
**• In abstract, last meaning of last sentence is not clear. With phrase “use of medication adherently lowers the odds of uncontrolled blood pressure (aOR: 0.21; 47 95% CI: 0.14-0.38)” do author mean to say ‘adherence to medication lowers the odds’?**

Thank you, reviewer, for accurately pointing out the mistake. In the revised manuscript we have revised the sentence as this “*adherence to antihypertensive medication lowered the odds of uncontrolled blood pressure (aOR: 0.21; 95% CI: 0.14–0.38).*”

**• Authors have used ‘uncontrolled blood pressure’ in some places and ‘uncontrolled hypertension’ in other instances. It is recommended that authors maintain consistency in use of terms. Authors are recommended to stick with the terms used in operational definition of the methodology section. Similarly, authors have used ‘elevated blood pressure’ in some parts of manuscript and ‘high blood pressure’ in other. Unless these mean different things, it is recommended to maintain consistency in terms we use. Further, authors have used term ‘compliance’ in operational definition and ‘adherence in some other places. This also needs correction.**

Thank you, reviewer, for accurately identifying the inconsistencies in terminology. We have carefully reviewed the manuscript and ensured consistency in the use of terms related to blood pressure; wherever the meaning was the same, we have replaced the terms accordingly to align with the operational definitions provided in the Methods section.

Regarding the use of “compliance” and “adherence,” the terminology reflects the wording used in the respective measurement scales. Specifically, “compliance” is used as stated in the Hill-Bone Compliance Scale, while “adherence” is used for the medication adherence domain within the same scale. Similarly, the term “drug compliance” is applied as defined in the Hypertension Knowledge-Level Scale (HKLS). Although the terms differ, their usage corresponds directly to the original scale constructs, and we have ensured that this distinction is clearly maintained in the manuscript.

**• Authors should check the abbreviations in the manuscript. For example, towards last part of introduction sections, authors have used ‘DALY’ without presenting the full form (line number 83). Similarly, BMI, previously abbreviated in methods and materials appears with full form in discussion section.**

Thank you, reviewer, for pointing this out. We have carefully reviewed the manuscript to ensure consistency in the use of abbreviations. All abbreviations, including “DALY” and “BMI,” have now been defined at their first mention and used consistently throughout the manuscript.

**• In line number 87, include space between ‘include’ and ‘non’**

Thank you, reviewer for pointing this out. We have revised the manuscript accordingly.

• **In line number 118, expand a bit about RE-AIM framework and the original study.**

Thank you for the suggestion. We have expanded the description in line 118 to provide more context about the RE-AIM framework and the original study. Specifically, we now explain that RE-AIM evaluates interventions across five dimensions: **Reach** (proportion of the target population engaged), **Effectiveness** (impact on health outcomes), **Adoption** (uptake by implementers), **Implementation** (fidelity and consistency), and **Maintenance** (sustainability over time). We also clarify that the original FCHV-led study applied this framework to implement and evaluate a community-based hypertension prevention and control strategy in Namobuddha Municipality, focusing on both intervention effectiveness and feasibility in a predominantly rural setting.

In revised manuscript, it has been mentioned as follow : “*We analyzed population-based baseline cross-sectional data collected between August and November 2023 in Namobuddha Municipality to assess uncontrolled blood pressure as part of a Female Community Health Volunteer (FCHV)–led implementation study guided by the RE-AIM (reach, effectiveness, adoption, implementation, and maintenance) framework [23,24]. The RE-AIM framework evaluates interventions across five key dimensions: Reach (the proportion of the target population engaged), Effectiveness (impact on outcomes), Adoption (uptake by implementers), Implementation (fidelity and consistency), and Maintenance (sustainability over time). While the baseline data focus on estimating the prevalence of uncontrolled blood pressure, the original study aims to evaluate the implementation outcomes of the FCHV-led hypertension prevention and control strategy in Namobuddha municipality . Although Namobuddha is administratively classified as a municipality, it retains predominantly rural characteristics—such as dispersed settlements, limited health service availability, and agriculture-based livelihoods—making it comparable to other rural communities in Nepal [25].”*

**• In line number 120, the study setting is not adequately justified to represent the rural setting. Municipality are considered urban setting unless classified through rigorous assessment to some rural category. Authors can borrow assessment from Degree of Urbanization report to justify the study setting as rural.**

Thank you, reviewer, for carefully reviewing this statement and suggesting the citation of the Degree of Urbanization report. In accordance with your suggestion, we have cited and referenced the report to justify the study setting as predominantly rural, despite Namobuddha being administratively classified as a municipality. The report supports that the area retains rural characteristics, including dispersed settlements, limited health services, and agriculture-based livelihoods, making it comparable to other rural communities in Nepal.

**• In line number 132, authors state that those confirmed as hypertensive from nearest health facility were considered for the study. What if the health facility confirming was not nearest to the residence of participants? Some people may have been confirmed from Kathmandu, Dhulikhel which may not be the nearest one.**

Thank you for your comment and query. Participants were advised to confirm their hypertension status at a health facility for easier access; however, we included all participants regardless of where their hypertension was confirmed, including those diagnosed at facilities farther from their residence, such as in Kathmandu or Dhulikhel. To reflect this, we have deleted the word “nearest” in the revised manuscript.

• **Authors have stated ‘well-trained interviewer’ conducted the interview. Rather than using judgemental words like well trained, authors should mention briefly the duration of training and contents covered during the train. This makes the statement more neutral.**

Thank you for your suggestion. In the revised manuscript, we have replaced the phrase “well-trained interviewer” with a neutral description specifying the training provided. In revised manuscript, we have included the following sentences “*Interviewers received 2 days of training covering data collection techniques, face-to-face interview methods, and the use of data collection tools based on a modified standardized questionnaire adapted from the WHO STEPS survey (version 3.2) in Nepali*.” This provides a clear and transparent description of interviewer preparation without using subjective terms.

• **In line number 150, authors have stated current tobacco use is defined as “current smokers (those who had smoked cigarettes within the past 30 days), past smokers (those who had smoked in the past but had quit and did not currently smoke), and non-smokers (those who had never smoked). However, smoked tobacco is only one form of tobacco use. Considering the results section, it appears authors have considered smoking status of the participants. If it is the case, authors should clearly define that in methods section.**

Thank you for your comment. We would like to clarify that the classification in line 150 refers specifically to **current smokers**, not current tobacco use in general. We have revised the Methods section to explicitly define **smoking status**—current smokers, past smokers, and non-smokers—based on cigarette use, to avoid any confusion with other forms of tobacco consumption.
**• In line 152 to 157, authors have presented operational definition of alcohol users. However definitions seem confusing. Which category does a person fall if he consumed alcohol in last 12 months but not particularly in last 30 days.**

Thank you for your comment, which helped us recognize the lack of clarity in the previous labels. We have now revised the classification of alcohol consumption as follows:

- **Abstainers:** Individuals who have never consumed alcohol in their lifetime.
- **Non-current drinkers:** Individuals who consumed alcohol in the past or during the last 12 months but **not in the past 30 days**.
- **Current drinkers:** Individuals who consumed alcohol at least once in the past 30 days.

In revised manuscript we have revised as follow : “*We classified alcohol consumption into abstainers (those who had never consumed alcohol in their lifetime ), non-current drinkers (consumed alcohol in the past but did not drink in the last 30 days (including those who drank within the past 12 months but not in the past 30 days)), and current drinkers (those who had consumed alcohol at least once in the past 30 days).*”

This revised classification ensures clarity and makes it easy to understand.

**• In Table authors report GDR score but have not specified which specific measure of average is reported. It is not clear if it is mean and SD or median and IQR.**

Thank you for your comment. We apologize for the oversight. In the revised manuscript, we have clarified that the GDR score is reported as **mean ± standard deviation (SD)**.

**• Authors have used ‘less than primary’ in some places and ‘basic education’ in other parts. If authors meant to refer to same thing, they need to maintain consistency in use of terms.**

Thank you for pointing this out. We have carefully reviewed the manuscript and ensured consistency in terminology. All instances now use **“basic education”** to refer to participants with education below the primary level, in line with the operational definitions provided in the Methods section

With regards,

Bihungum Bista

Corresponding coauthor
