# Supplementary material for: The prevalence and associated factors of uncontrolled blood pressure in a rural community of Nepal: A cross-sectional study
Source: PLOS Glob Public Health. 2026 Jan 8;6(1):e0005301. doi: 10.1371/journal.pgph.0005301 (PMC12782391; doi:10.1371/journal.pgph.0005301)
Supplement: Peer review history

# Peer review: Decision Letter 0

**DOI:** 10.1371/journal.pgph.0005301.r001

**Authors:** Dinesh Bhandari

6 Aug 2025

PGPH-D-25-01683

The prevalence and associated factors of uncontrolled hypertension in a rural community of Nepal: A cross-sectional study

PLOS Global Public Health

Dear Dr. Bista,

Thank you for submitting your manuscript to PLOS Global Public Health. After careful consideration, we feel that it has merit but does not fully meet PLOS Global Public Health’s publication criteria as it currently stands. Therefore, we invite you to submit a revised version of the manuscript that addresses the points raised during the review process.

Please submit your revised manuscript by 04/09/2025. If you will need more time than this to complete your revisions, please reply to this message or contact the journal office at globalpubhealth@plos.org. When you're ready to submit your revision, log on to https://www.editorialmanager.com/pgph/ and select the 'Submissions Needing Revision' folder to locate your manuscript file.

Please include the following items when submitting your revised manuscript:

- A rebuttal letter that responds to each point raised by the editor and reviewer(s). You should upload this letter as a separate file labeled 'Response to Reviewers'.
- A marked-up copy of your manuscript that highlights changes made to the original version. You should upload this as a separate file labeled 'Revised Manuscript with Track Changes'.
- An unmarked version of your revised paper without tracked changes. You should upload this as a separate file labeled 'Manuscript'.

Guidelines for resubmitting your figure files are available below the reviewer comments at the end of this letter.

We look forward to receiving your revised manuscript.

Kind regards,

Dinesh Bhandari, Ph.D.

Academic Editor

PLOS Global Public Health

Journal Requirements:

If the reviewer comments include a recommendation to cite specific previously published works, please review and evaluate these publications to determine whether they are relevant and should be cited. There is no requirement to cite these works unless the editor has indicated otherwise. 

Additional Editor Comments (if provided):

Reviewers' comments:

Reviewer's Responses to Questions

**Comments to the Author**

1. Does this manuscript meet PLOS Global Public Health’s publication criteria?

Reviewer #1: Yes

Reviewer #2: Yes

**********

2. Has the statistical analysis been performed appropriately and rigorously?

Reviewer #1: Yes

Reviewer #2: Yes

**********

3. Have the authors made all data underlying the findings in their manuscript fully available (please refer to the Data Availability Statement at the start of the manuscript PDF file)?

The PLOS Data policy

Reviewer #1: Yes

Reviewer #2: Yes

**********

4. Is the manuscript presented in an intelligible fashion and written in standard English?

Reviewer #1: Yes

Reviewer #2: Yes

**********

Reviewer #1: • In the Nepalese context, municipalities are generally classified as urban areas. However, the authors have conducted this study in Namobuddha Municipality to generate evidence relevant to rural settings. The authors need to justify classifying this setting as rural or revise the title, setting description, and relevant methodological details accordingly.

• In the third sentence of the introduction, the authors state: “Each incremental increase in systolic blood pressure (SBP) by 20 mmHg or more than 115 mmHg, or diastolic blood pressure (DBP) by 10 mmHg or more than 75 mmHg, doubles the risk of experiencing cardiovascular events and major stroke for 40-89 years.” It seems the intended meaning is that each 20 mmHg increase above 115 mmHg (SBP) and 10 mmHg increase above 75 mmHg (DBP) doubles the risk. However, the current phrasing is unclear and needs revision.

• In lines 79 to 82, the authors shift abruptly from discussing cost-effective interventions for NCDs to identifying hypertension as a major public health challenge. It would be clearer to focus the entire sentence specifically on hypertension.

• The authors mention conducting screening camps at the community level, intending to screen all individuals visiting the health facilities regardless of BP level. However, they specify BP thresholds in the eligibility criteria. If screening was conducted among all visitors but the analysis was limited to those meeting the eligibility criteria, the text should be revised accordingly. For instance: “Screening was conducted among all participants, but this analysis is confined to 1,008 participants meeting the eligibility criteria.”

• In line 139, the authors list both ‘married’ and ‘ever married’ as marital status categories. These are overlapping, as ‘married’ falls under ‘ever married.’ This should be corrected. If ‘ever married’ is intended to refer to divorced or separated individuals, it should be specified clearly.

• The phrase “Physical measurements included the following components” is unnecessary, as the measurements are listed sequentially and have not been categorized in earlier sections. This sentence can be removed.

• In Table 1, the percentage symbol (%) is not needed in the last column under the ethnicity variable and should be removed.

• In line 221, the authors state that 81.5% of new cases were uncontrolled, implying that the remaining were controlled. However, it is unclear how 'new cases' were defined. Individuals with controlled BP and no prior diagnosis would be considered normotensive, not new cases. This needs clarification.

• Also in line 221, the authors mention that “healthy behaviours were sub-optimal.” Unless an index variable for ‘healthy behaviour’ was created, this sentence should be omitted. The authors can instead present the findings for individual behavioural variables.

• In Table 2, the authors should specify that the numbers represent mean and standard deviation. This can be done using parentheses alongside each variable name (e.g., “Age (mean ± SD)”).

• It appears that some cell values are limited due to small sample sizes under educational status in Table 2. Unless clinically justified, the authors may consider merging 'secondary' and 'bachelor’s and above' into a single category: ‘secondary and above.’

• In Table 2, the category ‘in last 12 months’ under alcohol intake is not self-explanatory. To improve clarity, it should be revised to ‘intake in the last 12 months.’

• A thorough grammar check is recommended throughout the manuscript.

Reviewer #2: Thank you for the opportunity to review the paper. This is an important paper. However, I have following suggestion to improve rigor and quality of the paper:

1. Can you explain the sampling technique for this study?

2. What is the definition of uncontrolled hypertension? The paper at the moment looks like an analysis of uncontrolled blood pressure rather than the prevalence of uncontrolled hypertension prevalence. I presume they are different given uncontrolled hypertension considers persistent raised BP even after pharmacological or lifestyle intervention.

3. Detailed baseline characteristics of the missing and excluded cases are needed in the result section. Was there any difference in their characteristics?

4. Can you also specify the mean blood pressure by age group?

5. It is also unclear if the paper is focusing on lifestyle medication or pharmacological control of hypertension.

6. How are new cases assessed for medication adherence? What is the definition of new cases?

7. The cut off points and definition for hypertension differs in line 123- 126, and line 186: Blood pressure measurement.

8. Need references to this statement: In resource-limited rural settings, 295 public health interventions that promote healthy eating and physical activity—delivered through 296 existing community structures such as Female Community Health Volunteers (FCHVs)—could 297 help address this modifiable risk factor

9. Enrolment into Medication and medication adherence are two different variables. Simply enrolling in medication can be a predictor for blood pressure control. So, would suggest to disintegrate as two variables after the collinearity check

10. I would recommend a thorough review of the term “uncontrolled hypertension” as this paper basically focuses on the prevalence of hypertension or “uncontrolled blood pressure”. The discussion section should be appropriated accordingly.

**********

what does this mean? ). If published, this will include your full peer review and any attached files.

**Do you want your identity to be public for this peer review?** If you choose “no”, your identity will remain anonymous but your review may still be made public.

For information about this choice, including consent withdrawal, please see our Privacy Policy

Reviewer #1: No

Reviewer #2: No

**********

[NOTE: If reviewer comments were submitted as an attachment file, they will be attached to this email and accessible via the submission site. Please log into your account, locate the manuscript record, and check for the action link "View Attachments". If this link does not appear, there are no attachment files.]

While revising your submission, please upload your figure files to the Preflight Analysis and Conversion Engine (PACE) digital diagnostic tool, https://pacev2.apexcovantage.com/. PACE helps ensure that figures meet PLOS requirements. To use PACE, you must first register as a user. Registration is free. Then, login and navigate to the UPLOAD tab, where you will find detailed instructions on how to use the tool. If you encounter any issues or have any questions when using PACE, please email PLOS at figures@plos.org. Please note that Supporting Information files do not need this step.

# Author comment: Author response to Decision Letter 1

**DOI:** 10.1371/journal.pgph.0005301.r002

23 Sep 2025

Attachment Submitted filename: Response to Reviewers.docx

# Peer review: Decision Letter 1

**DOI:** 10.1371/journal.pgph.0005301.r003

**Authors:** Dinesh Bhandari

19 Nov 2025

PGPH-D-25-01683R1

The prevalence and associated factors of uncontrolled blood pressure in a rural community of Nepal: A cross-sectional study

PLOS Global Public Health

Dear Dr. Bista,

Thank you for submitting your manuscript to PLOS Global Public Health. After careful consideration, we feel that it has merit but does not fully meet PLOS Global Public Health’s publication criteria as it currently stands. Therefore, we invite you to submit a revised version of the manuscript that addresses the points raised during the review process.

Please submit your revised manuscript by 10/12/2024. If you will need more time than this to complete your revisions, please reply to this message or contact the journal office at globalpubhealth@plos.org. When you're ready to submit your revision, log on to https://www.editorialmanager.com/pgph/ and select the 'Submissions Needing Revision' folder to locate your manuscript file.

Please include the following items when submitting your revised manuscript:

- A rebuttal letter that responds to each point raised by the editor and reviewer(s). You should upload this letter as a separate file labeled 'Response to Reviewers'.
- A marked-up copy of your manuscript that highlights changes made to the original version. You should upload this as a separate file labeled 'Revised Manuscript with Track Changes'.
- An unmarked version of your revised paper without tracked changes. You should upload this as a separate file labeled 'Manuscript'.

Guidelines for resubmitting your figure files are available below the reviewer comments at the end of this letter.

We look forward to receiving your revised manuscript.

Kind regards,

Dinesh Bhandari, Ph.D.

Academic Editor

PLOS Global Public Health

Journal Requirements:

If the reviewer comments include a recommendation to cite specific previously published works, please review and evaluate these publications to determine whether they are relevant and should be cited. There is no requirement to cite these works unless the editor has indicated otherwise. 

Please review your reference list to ensure that it is complete and correct. If you have cited papers that have been retracted, please include the rationale for doing so in the manuscript text, or remove these references and replace them with relevant current references. Any changes to the reference list should be mentioned in the rebuttal letter that accompanies your revised manuscript. If you need to cite a retracted article, indicate the article’s retracted status in the References list and also include a citation and full reference for the retraction notice.

Additional Editor Comments (if provided):

[Note: HTML markup is below. Please do not edit.]

Reviewers' comments:

Reviewer's Responses to Questions

**Comments to the Author**

Reviewer #3: All comments have been addressed

**********

publication criteria?

Reviewer #3: Yes

**********

3. Has the statistical analysis been performed appropriately and rigorously?

Reviewer #3: Yes

**********

4. Have the authors made all data underlying the findings in their manuscript fully available (please refer to the Data Availability Statement at the start of the manuscript PDF file)?

The PLOS Data policy

Reviewer #3: Yes

**********

5. Is the manuscript presented in an intelligible fashion and written in standard English?

Reviewer #3: Yes

**********

Reviewer #3: Feedback

• In abstract, last meaning of last sentence is not clear. With phrase “use of medication adherently lowers the odds of uncontrolled blood pressure (aOR: 0.21; 47 95% CI: 0.14-0.38)” do author mean to say ‘adherence to medication lowers the odds’?

• Authors have used ‘uncontrolled blood pressure’ in some places and ‘uncontrolled hypertension’ in other instances. It is recommended that authors maintain consistency in use of terms. Authors are recommended to stick with the terms used in operational definition of the methodology section. Similarly, authors have used ‘elevated blood pressure’ in some parts of manuscript and ‘high blood pressure’ in other. Unless these mean different things, it is recommend to maintain consistency in terms we use. Further, authors have used term ‘compliance’ in operational definition and ‘adherence in some other places. This also needs correction.

• Authors should check the abbreviations in the manuscript. For example, towards last part of introduction sections, authors have used ‘DALY’ without presenting the full form (line number 83). Similarly, BMI, previously abbreviated in methods and materials appears with full form in discussion section.

• In line number 87, include space between ‘include’ and ‘non’

• In line number 118, expand a bit about RE-AIM framework and the original study.

• In line number 120, the study setting is not adequately justified to represent the rural setting. Municipality are considered urban setting unless classified through rigorous assessment to some rural category. Authors can borrow assessment from Degree of Urbanization report to justify the study setting as rural.

• In line number 132, authors state that those confirmed as hypertensive from nearest health facility were considered for the study. What if the health facility confirming was not nearest to the residence of participants? Some people may have been confirmed from Kathmandu, Dhulikhel which may not be the nearest one.

• Authors have stated ‘well-trained interviewer’ conducted the interview. Rather than using judgemental words like well trained, authors should mention briefly the duration of training and contents covered during the train. This makes the statement more neutral.

• In line number 150, authors have stated current tobacco use is defined as “current smokers (those who had smoked cigarettes within the past 30 days), past smokers (those who had smoked in the past but had quit and did not currently smoke), and non-smokers (those who had never smoked). However, smoked tobacco is only one form of tobacco use. Considering the results section, it appears authors have considered smoking status of the participants. If it is the case, authors should clearly define that in methods section.

• In line 152 to 157, authors have presented operational definition of alcohol users. However definitions seem confusing. Which category does a person fall if he consumed alcohol in last 12 months but not particularly in last 30 days.

• In Table authors report GDR score but have not specified which specific measure of average is reported. It is not clear if it is mean and SD or median and IQR.

• Authors have used ‘less than primary’ in some places and ‘basic education’ in other parts. If authors meant to refer to same thing, they need to maintain consistency in use of terms.

**********

what does this mean? ). If published, this will include your full peer review and any attached files.

**Do you want your identity to be public for this peer review?** If you choose “no”, your identity will remain anonymous but your review may still be made public.

For information about this choice, including consent withdrawal, please see our Privacy Policy

Reviewer #3: No

**********

[NOTE: If reviewer comments were submitted as an attachment file, they will be attached to this email and accessible via the submission site. Please log into your account, locate the manuscript record, and check for the action link "View Attachments". If this link does not appear, there are no attachment files.]

# Author comment: Author response to Decision Letter 2

**DOI:** 10.1371/journal.pgph.0005301.r004

11 Dec 2025

Attachment Submitted filename: Response_to_Reviewers_auresp_2.docx

# Editor report: Decision Letter 2

**DOI:** 10.1371/journal.pgph.0005301.r005

**Authors:** Dinesh Bhandari

18 Dec 2025

The prevalence and associated factors of uncontrolled blood pressure in a rural community of Nepal: A cross-sectional study

PGPH-D-25-01683R2

Dear Dr Bista,

We are pleased to inform you that your manuscript 'The prevalence and associated factors of uncontrolled blood pressure in a rural community of Nepal: A cross-sectional study' has been provisionally accepted for publication in PLOS Global Public Health.

Before your manuscript can be formally accepted you will need to complete some formatting changes, which you will receive in a follow up email. A member of our team will be in touch with a set of requests.

Please note that your manuscript will not be scheduled for publication until you have made the required changes, so a swift response is appreciated.

IMPORTANT: The editorial review process is now complete. PLOS will only permit corrections to spelling, formatting or significant scientific errors from this point onwards. Requests for major changes, or any which affect the scientific understanding of your work, will cause delays to the publication date of your manuscript.

If your institution or institutions have a press office, please notify them about your upcoming paper to help maximize its impact. If they'll be preparing press materials, please inform our press team as soon as possible -- no later than 48 hours after receiving the formal acceptance. Your manuscript will remain under strict press embargo until 2 pm Eastern Time on the date of publication. For more information, please contact globalpubhealth@plos.org.

Thank you again for supporting Open Access publishing; we are looking forward to publishing your work in PLOS Global Public Health.

Best regards,

Dinesh Bhandari, Ph.D.

Academic Editor

PLOS Global Public Health

***********************************************************

Reviewer Comments (if any, and for reference):
